# Protein language models uncover carbohydrate-active enzyme function in metagenomics

**DOI:** 10.1186/s12859-025-06286-y

**Published:** 2025-11-26

**Authors:** Kumar Thurimella, Ahmed M. T. Mohamed, Chenhao Li, Tommi Vatanen, Daniel B. Graham, Róisín M. Owens, Sabina Leanti La Rosa, Damian R. Plichta, Sergio Bacallado, Ramnik J. Xavier

**Affiliations:** 1 Broad Institute of MIT and Harvard, Cambridge, MA USA; 2 Center for Computational and Integrative Biology and Department of Molecular Biology, Massachusetts General Hospital, Harvard Medical School, Boston, MA USA; 3 Institute of Biotechnology, Helsinki Institute of Life Science, University of Helsinki, Helsinki, Finland; 4 Department of Microbiology, Faculty of Agriculture and Forestry, University of Helsinki, Helsinki, Finland; 5 Research Program for Clinical and Molecular Metabolism, Faculty of Medicine, University of Helsinki, Helsinki, Finland; 6 Liggins Institute, University of Auckland, Auckland, New Zealand; 7 Department of Chemical Engineering and Biotechnology, University of Cambridge, Cambridge, UK; 8 School of Medicine, University of Colorado Anschutz Medical Campus, Aurora, CO USA; 9 Faculty of Chemistry, Biotechnology and Food Science, Norwegian University of Life Sciences, Ås, Norway; 10 Department of Pure Mathematics and Mathematical Statistics, University of Cambridge, Cambridge, UK

**Keywords:** Protein language models, Deep learning, CAZymes, Fibrosis, Crohn’s disease, IgG4-related disease, Systemic sclerosis

## Abstract

**Background:**

The functional annotation of uncharacterized microbial enzymes from metagenomic data remains a significant challenge, limiting our understanding of microbial metabolic dynamics. Traditional annotation methods often rely on sequence homology, which can fail to identify remote homologs or enzymes with structural rather than sequence conservation. To address this gap, we developed CAZyLingua, the first annotation tool to use protein language models (pLMs) for the accurate classification of carbohydrate-active enzyme (CAZyme) families and subfamilies.

**Results:**

CAZyLingua demonstrated high performance, maintaining precision and recall comparable to state-of-the-art hidden Markov model-based methods while outperforming purely sequence-based approaches. When applied to a metagenomic gene catalog from mother/infant pairs, CAZyLingua identified over 27,000 putative CAZymes missed by other tools, including horizontally-transferred enzymes implicated in infant microbiome development. In datasets from patients with Crohn’s disease and IgG4-related disease, CAZyLinuga uncovered disease-associated CAZymes, highlighting an expansion of carbohydrate esterases (CEs) in IgG4-related disease. A CE17 enzyme predicted to be overabundant in Crohn’s disease was functionally validated, confirming its catalytic activity on acetylated manno-oligosaccharides.

**Conclusions:**

CAZyLingua is a powerful tool that effectively augments existing functional annotation pipelines for CAZymes. By leveraging the deep contextual information captured by pLMs, our method can uncover novel CAZyme diversity and reveal enzymatic functions relevant to health and disease, contributing to a further understanding of biological processes related to host health and nutrition.

**Supplementary Information:**

The online version contains supplementary material available at 10.1186/s12859-025-06286-y.

## Background

Rapid advancements in sequencing technologies have led to an abundance of genomic data, outpacing the capacity to annotate and decipher the functions of these sequences [1]. A significant challenge arises in contextualizing the vast number of unknown functions present in microbes [2, 3] and, as a consequence, a substantial fraction of microbial proteins remains unannotated [4–6]. The Unified Human Gastrointestinal Protein (UHGP) catalog alone holds greater than 170 million protein sequences, of which 40% lack any functional annotation [2]. Elucidating the function of these sequences has the potential to provide insights into microbial metabolic behaviors and niches within a particular ecosystem, including the dynamics of microbial–host interactions [7–9].

In microbial genomics, accurate annotations of the biological functions of enzymes is critical, as these molecules have important roles in catalyzing essential biochemical reactions with high specificity and efficiency. [10–13]. Carbohydrate-active enzymes (CAZymes) play fundamental roles in various biological processes, including cell structure, signaling, energy storage, and nutrient processing [14–16]. Metagenomic sequencing and functional ‘omics have shown that CAZymes support the growth of beneficial microbes in infants by catabolizing human milk oligosaccharides (HMOs) [17, 18]. CAZymes have also been found to play a role in the microbiomes of patients with inflammatory diseases like Crohn’s disease (CD) [19] and IgG4-related disease (IgG4-RD), in which there is upregulation of glycan-related pathways [20].

Historically, functional annotation tools have relied on hidden Markov models (HMMs) [21, 22] that are built by aligning many amino acid sequences or using sequence homology tools like BLAST, which employs a pairwise alignment strategy between query and target sequences [23, 24]. The current state-of-the-art tool for annotating CAZymes, dbCAN2, similarly relies on sequence homology or HMMs [25]. While having achieved significant effectiveness in genomic sciences, these methods are not able to assign a biological role to one-third of all bacterial proteins [26]. Advancements in deep learning have significantly aided the functional annotation of proteins and comprehension of their diverse functions [27–34]. Protein language models (pLMs), such as those used for structural prediction and other tasks, demonstrate remarkable capabilities in decoding the intricate amino acid language of proteins, which facilitates their functional annotation through a distinct approach compared to sequence-based alignment methods [34–38]. CAZymes are classified into distinct classes of glycoside hydrolases (GHs), polysaccharide lyases (PLs), glycosyltransferases (GTs), and carbohydrate esterases (CEs). Within a class, the enzymes share a conserved fold, mechanism, and catalytic residues [15]. With this fine-grained ontology and a set of distinct enzymatic reactions, CAZymes represent an ideal training dataset for pLMs.

Here, we present CAZyLingua, the first annotation tool to harness pLMs for the accurate classification of CAZymes. We applied CAZyLingua to gene catalogs derived from human microbiome metagenomic datasets and identified CAZymes implicated in health and disease states. Our first gene catalog was constructed from paired mother/infant metagenomes [39] consisting of ~ 2,000,000 proteins, from which we uncovered ~ 27,000 CAZymes previously undetected by dbCAN2 or eggNOG. Early persistence of diverse microbial strains in the gut has been linked with metabolic pathways utilizing CAZymes, including breakdown of HMOs and dietary polysaccharides and metabolism of mucin in the colon [40]. CAZyLingua was then applied to a metagenomic dataset derived from patients with inflammatory and fibrosis-prone diseases, including CD and IgG4-RD. We observed that a greater percentage of genes significantly less abundant in CD were predicted to be CAZymes, while in IgG4-RD, we found an expansion of hundreds of CEs in particular. We focused on CEs in CD and found a CE17 that was overabundant in the disease state. Using MALDI-ToF mass spectrometry, we validated the function of CE17 on the enzyme family’s known substrates, acetylated manno-oligosaccharides. We demonstrate that CAZyLingua achieves high model accuracy compared to standard sequence homology tools and can be used to augment the functional annotation of CAZymes in metagenomic studies, providing valuable insights into the diversity and functional potential of these microbial enzymes.

## Methods

### Overview of the CAZyLingua pipeline

**Fig. 1:**
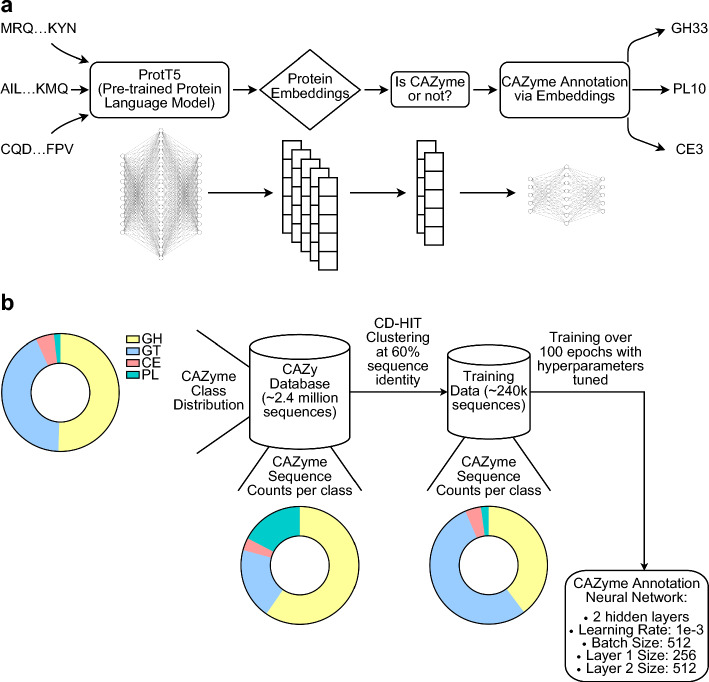
CAZyLingua: a deep learning model used for the classification of proteins as CAZymes. **a** The workflow of CAZyLingua starts with raw embeddings from ProtT5 followed by the use of those embeddings as input through two classifiers to distinguish (1) whether the embedding was a CAZyme and if so, (2) to which CAZyme family it belongs. **b** The training strategy for CAZyLingua began with a 60% sequence identity clustering to remove redundancy from the CAZy database in order to train on distinct CAZymes. The cross-entropy loss function was applied for training and the loss function that was used included a weighted balancing function to proportionally sample the number of representative sequences per CAZyme class/family/subfamily in the database. This strategy was employed so as not to oversample on highly represented families

The CAZyLingua pipeline is a multi-step tool designed for the functional annotation of CAZymes using pLMs (Fig. 1a). The workflow begins by generating numerical embeddings for amino acid sequences using the ProtT5 model. These embeddings serve as input for a two-stage classification process. First, a random forest (RF) classifier predicts whether a query sequence is a CAZyme. If the sequence is identified as a CAZyme, a second, multiclass neural network classifier annotates it with a specific CAZyme family or subfamily from the CAZy database ontology (Fig. 1b). The models were trained on curated datasets derived from the CAZy database and other protein catalogs and benchmarked against a hold-out set of gold-standard genomes, as detailed in the following sections.

### CAZyme training dataset curation

The CAZy database found at http://www.cazy.org/IMG/cazy_data/cazy_data.zip is cataloged by the dbCAN2 tool maintainers, and a fasta file is available at https://bcb.unl.edu/dbCAN2/download/. We downloaded the CAZy database as of August 06, 2022 containing 2,428,817 sequences as it was the latest version that was available when we began training the model. We chose to focus on the four main classes of CAZymes: 173 families and 177 subfamilies in GHs, 115 families in GTs, 20 families in CEs, and 42 families and 60 subfamilies in PLs. We removed everything that did not belong to one of these families and any sequences that were larger than 5,000 amino acids in length to prevent GPU out of memory errors when generating embeddings. The entire number of remaining sequences was 2,413,796: 1,221,013 in GH, 1,027,247 in GT, 122,413 in CE, and 43,123 in PL.

Using the CD-HIT software tool [41], we clustered our CAZy database at 60% sequence identity. CD-HIT returns a representative sequence for a given cluster. The clusters were created such that, in the resulting database (nr.CAZy.60.fasta), no two sequences had a sequence similarity greater than 60%. The resulting database preserved all of the original families and subfamilies while reducing the redundancy in the database. The database in nr.CAZy.60.fasta contained 232,736 sequences, of which 92,385 sequences were in GH, 125,240 in GT, 10,177 in CE, and 4,934 in PL.

Following the curation of the CAZy sequences, we used ProtT5 (in bio_embeddings v1.4) [37, 42] to generate embeddings for each of these sequences using a V100 GPU. We stored the embeddings in h5 files, following the hierarchical data format (HDF). This embedding database served as the training dataset for both classifiers in CAZyLingua.

### Random forest training and testing

To build the binary classifier for CAZyLingua, we curated comprehensive positive (CAZyme) and negative (non-CAZyme) training datasets. For the positive set, we used the entirety of the CAZy database from August 06, 2022. We removed duplicate sequences (100% sequence identity) using CD-HIT [41] and filtered out any sequences longer than 5,000 amino acids, resulting in a final set of 1,513,258 CAZyme sequences.

To construct a robust negative set, we combined protein sequences from several databases: the protein families database Pfam (version 32.0; 1,296,280 seed sequences), the Kyoto Encyclopedia of Genes and Genomes (KEGG; 3,435 non-CAZyme enzymes as of July 2022), and Swiss-Prot (967,449 proteins). To ensure the negative set was free of any potential CAZyme homologs, we performed a sensitive homology search using DIAMOND [43] (ultra-sensitive BLASTp setting) between our combined negative set and the CAZy database. Any protein from the negative set that registered a hit was removed.

Using the embeddings from these curated datasets, we trained a RF model for the binary classification task (CAZyme vs. non-CAZyme). We used the RandomForestClassifier class from the scikit-learn library (version 1.5.1) [44], setting the number of estimators to 200 and using a fixed integer for the random state to ensure reproducibility.

To evaluate the model’s performance on unseen data and across diverse taxa, we benchmarked it against a hold-out test set of 12 gold-standard bacterial genomes with manually curated annotations. Initial benchmarking focused on three species selected for their biological relevance and varying proportions of CAZymes: *Bacteroides thetaiotaomicron* (7.6% CAZymes), *Eggerthella lenta* (1.1%), and *Ruminococcus gnavus* (3.0%). Any protein sequence with 100% identity to a sequence in our test set was removed from the training data. The model’s performance metrics (precision, recall, F1 score) on these 12 species are detailed in Additional file 1: Table S1.

### Feed forward neural network architecture

The final stage in the CAZyLingua model is the multiclass classification for a given CAZyme family based on the embeddings selected as CAZyme from the RF. The feedforward neural network architecture has three overall layers with two hidden layers. The fixed size input of 1024 dimensions from ProtT5 embeddings are projected to 256 dimensions then to 512 dimensions to a final classification output layer of 574, which reflects the number of CAZyme families and subfamilies. We implemented this model using Pytorch Lightning [45] to create a classifier that included all of the training, validation, and testing steps. A post-hoc analysis of the model’s weights revealed no meaningful relationship between any specific index position in the input embedding and the final classification of a sequence as a CAZyme (Additional file 1: Fig. S1).

The model used a cross-entropy loss from PyTorch [46] with the weights parameter set to balance the number of sequences from the different families and subfamilies. To prevent overtraining on highly represented families, the loss function penalty for a given family was calculated as the inverse of the number of sequences per family. This ensures that if the model is incorrectly labeling a family with very few training examples there will be a stronger penalty in comparison to incorrectly labeling a family with a higher proportion of the training examples.

### Hyperparameter optimization and neural network training

The multiclass classification neural network in the CAZyLingua pipeline was trained using RayTune [47], a hyperparameter tuning library. The hyperparameters that were tested were the size of layer 1, the size of layer 2, the batch size, and the learning rate. To find the optimal hyperparameters to select the most accurately trained model, 20 models were tested in parallel with random sampled hyperparameters selected by RayTune (Additional file 1: Table S2). Each model was trained over 100 epochs using the Async Successive Halving (ASHA) [48] scheduler that terminates a model (early stopping) optimized to minimize the training loss. Metrics for the validation accuracy were collected after each epoch, and the testing accuracy was collected after the model was fully trained. Each training model was visualized using TensorBoard [49] (Additional file 1: Fig. S2).

### General embedding generation and technical performance details

We generated embeddings using the bio_embeddings library ($bio_embeddings config.yml) custom built into a Terra (https://terra.bio/) workflow. The config file was specified to use the ProtT5 embedder. Each virtual machine used to generate embeddings had 1 nvidia-tesla-p100 GPU, 16 vCPU, 32 GB of RAM, and 100 GB boot disk. The time to generate 2,670 protein embeddings took 4 min and 2 s with these specifications. The training time for the RF classifier was 49 min and 38 s using a virtual machine with 64 vCPUs and 128 GB of memory. For the training of the CAZyme family annotation tool, the training took place over 6 h and 37 min on a virtual machine with 1 nvidia-tesla-p100 GPU, 12vCPU, and 85 GB of memory.

To run the tool on the marimo web application took 48 s on 2,670 protein embeddings. The loading of both models into the application took 31 s.

### Benchmarking of CAZyme/non-CAZyme RF classifier

To benchmark the RF classifier, we used different metrics to quantify the performance of CAZyLingua to dbCAN2. We ensured that our test genomes were not in the training set by removing any 100% identical sequences in the training set, a procedure laid out by dbCAN2 [25]. For the F1 score, we followed a standard formula:

$$ {\text{F}}1\;{\text{Score}} = 2 \times \frac{{{\text{recall}} \times {\text{precision}}}}{{{\text{recall}} + {\text{precision}}}} $$

where we define recall and precision as follows:

$$ {\text{Precision}} = \frac{{{\text{True}}\;{\text{Positives}}}}{{{\text{True}}\;{\text{Positives}} + {\text{False}}\;{\text{Positives}}}} $$

$$ {\text{Recall}} = \frac{{{\text{True}}\;{\text{Positives}}}}{{{\text{True}}\;{\text{Positives}} + {\text{False}}\;{\text{Negatives}}}} $$

The precision-recall and ROC curves were plotted using scikit-learn [44] using the precision_recall_curve and roc_curve using the e-values from dbCAN2 and the scores from the decision function of the RF from CAZyLingua as the target scores.

**Fig. 2:**
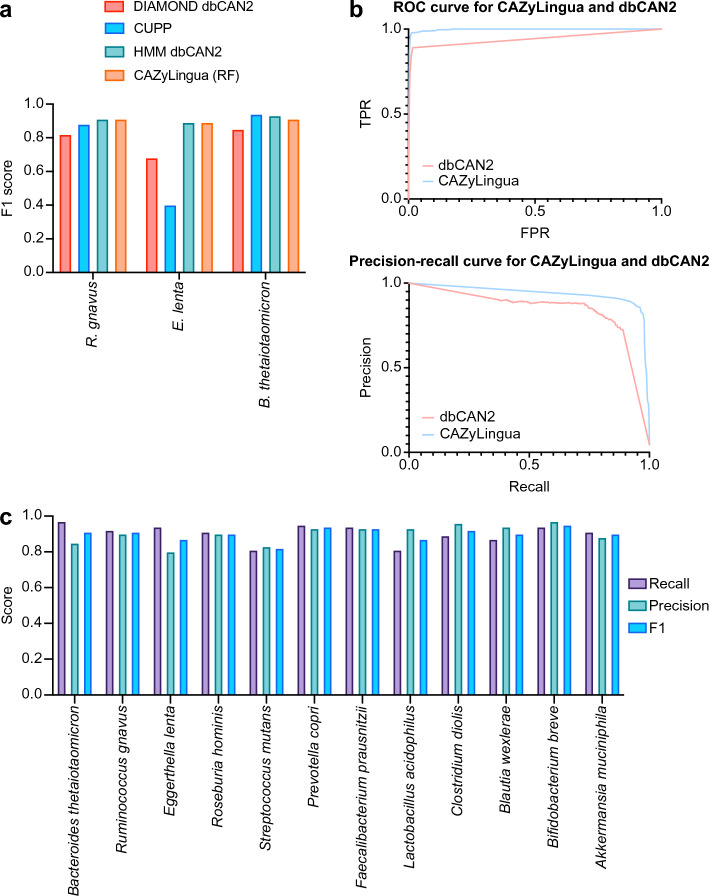
CAZyLingua performance relative to the BLAST-based CAZyme annotation tool dbCAN2. The final CAZyLingua Random Forest (RF) model was benchmarked using gold-standard annotated genomes. **a** Head-to-head comparison of F1 scores for CAZyLingua (RF), dbCAN2 (HMM and DIAMOND), and CUPP on three initial test genomes. **b** ROC (top) and precision-recall (bottom) curves comparing the CAZyLingua RF classifier against the dbCAN2 HMM on predictions from the three benchmarked genomes, with the genomes held out of the training data. The RF model’s prediction probabilities and the dbCAN2 DIAMOND e-values were used as the respective target scores for generating the curves. **c** Performance of the CAZyLingua RF model across an expanded set of 12 gold-standard bacterial genomes, demonstrating robust precision, recall, and F1 scores across diverse taxa

We additionally benchmarked our RF model from CAZyLingua and compared them alongside the HMM and DIAMOND modules in dbCAN2 [25] as well as CUPP [50], with the results shown in Fig. 2a and Additional file 1: Table S3. The classification_report function was used to generate the precision, recall, and F1 scores, and the confusion_matrix function was used to generate the confusion matrix. Both of these functions were used from scikit-learn (version 1.5.1) [44].

### Gene catalog construction

The metagenomes for each disease type (IgG4-RD [20] and CD [51]) and for the mother/infant cohort [39] were assembled into their respective gene catalogs following the same procedure. A quality control check was performed using Trim Galore! [52] to remove sequencing adapters and kneadData to remove human reads and trim low-quality reads (–trimmomatic-options “HEADCROP:15 SLIDINGWINDOW:1:20 MINLEN:50”) to keep reads that were a minimum of 50 bp long. All the quality controlled reads were assembled using MEGAHIT [53]. Each contig had all of the open readings frames predicted using Prodigal [54], and we kept both gene and protein sequences. A non-redundant gene catalog was built with a sequence identity threshold of 95% using CD-HIT [41]. To construct a count matrix, each read was mapped using a Burrows-Wheeler Aligner with at least 95% sequence identity for the length of the read. To determine the taxonomy of each contig, MMseqs2 [55] was used with NCBI RefSeq as the taxonomic annotation database post-hoc, since CAZyLingua does not provide taxonomic assignments.

The IgG4-RD non-redundant (90% sequence identity) gene catalog consisted of 2,237,319 genes from 58 IgG4-RD samples and 165 healthy control samples [20]. The CD non-redundant (90% sequence identity) gene catalog consisted of 5,929,528 genes from 68 CD samples and 34 non-IBD control samples [51]. The mother/infant non-redundant (95% sequence identity) gene catalog consisted of 2,327,970 genes, with 74 infants, 137 mothers, and 70 mother/infant pairs. Infants were sampled each month between birth (0 months) and 12 months (and additionally at 0.5 months), and mothers were sampled at gestational week 27 (approximately 3 months prior to the birth of the child) and at 3, 6, 9, and 12 months after the birth [39]. Each of these gene catalogs was constructed in each respective prior study and directly utilized in the analysis presented in this paper.

### Analysis of mother/infant gene catalog

The entire mother/infant gene catalog was run through dbCAN2 (diamond blastp -d ${CAZy_reference} -q ${query_file} -o ${output_str}.matches.tsv -e 1e-102 -k 1 -p 2 -f 6) and eggNOG (version 2.1.12) on default parameters. Additionally, embeddings were generated for the entire mother/infant dataset using ProtT5, with CAZyLingua running inference on the entire gene catalog.

We took a 977 horizontally-transferred gene subset and collected all of the dbCAN2 and CAZyLingua results. There were 12 genes that only CAZyLingua predicted, and we performed a structural prediction on each of the protein sequences. We performed a Euclidean distance search between those 12 embeddings and the nr.CAZy.60.fasta database to find the closest embedding and subsequently the CAZyme family. We then used ColabFold [56] to fold each of the 12 proteins and their nearest neighbor to generate PDBs for each horizontally-transferred gene and neighbor pair. A structural alignment was computed on each of these pairs using Foldseek [57], which returns the overlapped structures and a TM score for each pair. To compute sequence homology metrics, we selected the “Align two or more sequences” option in the BLASTp suite on the NCBI website (https://blast.ncbi.nlm.nih.gov/Blast.cgi?PROGRAM=blastp%26PAGE_TYPE=BlastSearch%26LINK_LOC=blasthome).

The putative GH33 and each of the GH33 and GH43_13 in nr.CAZy.60.fasta were ordinated through tSNE (scikit-learn TSNE package) [44] and plotted using matplotlib [58]. A structural prediction of the putative GH33 was produced from ColabFold [56] and the amino acid residue substitution analysis was done using custom scripts. To search against known, experimentally-characterized structures, the DALI option to pairwise search against PDB25 [59] was used. To structurally align a pairwise hit from putative GH33 to a structure from PDB25, we used US-align [60] to generate aligned structures and a TM score.

### Disease metagenomic differential abundance analysis

In each disease gene catalog, linear modeling was used to regress different disease covariates onto each gene in the catalog to find differentially abundant genes (features). An abundance filter was applied to the entire count matrix to remove any genes with < 10% prevalence across samples. A zero-inflation was applied to any zeros in the count matrix, where the zero value would be replaced by the minimum non-zero value in the given feature and divided by 2. The fold change was calculated by dividing the mean of the disease group by the control group and taking the log_2_ of the value. Each value is log_2_ transformed, and a z-score is calculated for every value in a given feature using the scipy [61] library. A linear model, from the statsmodels [62] library, is then applied to each feature. For IgG4-RD, the metadata covariates modeled were: age, on treatment, rituximab, prednisone, other treatments, sex, and cohort. In CD, the variables modeled were: age, on antibiotics, mesalamine, and steroids. A significance threshold was established for all of the analyses: we followed a multiple testing adjustment, and p-values were corrected using Benjamini–Hochberg correction, with a false discovery rate (FDR)-corrected p-value (q-value) of 0.25. The volcano plots were labeled based on conditional arguments for the CD and IgG4-RD metagenomic catalogs.

For CD, the criteria for the displayed labels were:

1. logFC > 0
2. logFC < 0 and *p*-value < 1 × 10^–8^
3. logFC <  − 4.5 and *p*-value < 1 × 10^–3^

For IgG4-RD, the criteria for the displayed labels were:

1. logFC > 2 and *p*-value < 1 × 10^–5^
2. logFC <  − 2 and *p*-value < 1 × 10^–3.5^
3. logFC > 3 and *p*-value < 1 × 10^–2.5^
4. logFC <  − 3.5 and *p*-value < 1 × 10^–2^

### Recombinant protein production

KTCE17 was produced by Genscript according to the manufacturer’s protocol. Briefly, the synthetic gene was introduced in pET-30a( +), and the was construct transformed into *Escherichia coli* BL21 STAR cells (Invitrogen). A single colony was inoculated into LB medium containing 50 mg/L kanamycin, and the culture was incubated at 37 °C at 200 rpm. Once cell density reached an optical density at 600 nm of 0.8, protein overexpression was induced with isopropyl b-D-thiogalactopyranoside (IPTG) to a final concentration of 500 mM. Recombinant protein production continued for 16 h at 16 °C, after which the cells were collected by centrifugation. KTCE17 was purified by immobilized-metal ion affinity chromatography (IMAC). Pure KTCE17 samples were stored in 50 mM Tris–HCl, 150 mM NaCl, 10% glycerol, pH 8.0. Protein purity was estimated over 90% as determined by sodium dodecyl sulfate–polyacrylamide gel electrophoresis (SDS–PAGE) analysis. Protein concentration was determined using the Bradford assay (Bio-Rad, Germany).

### Activity assays

An acetylated galacto-glucomannan (AcGGM) digest was produced in-house using the endo-mannanase *Ri*GH26 [63] to produce a substrate containing de- and acetylated tetramers, pentamers, and hexamers (referred to as AcGGMoS4-5–6). Enzyme reactions contained 10 mM sodium phosphate (pH 5.8) and 0.1 mg/mL substrate. Reaction mixtures were pre-heated (37 °C for 10 min) in a Thermomixer C incubator (Eppendorf) before addition of KTCE17 to 1 mM (in a final reaction mixture volume of 100 µL) and incubated for 16 h at 37 °C and 700 rpm. Experiments were performed as two biological replicates. The previously characterized *Fp*CE17 [64] was used as a positive control.

### MALDI-ToF MS analysis of oligosaccharides

Reactions were analyzed by matrix-assisted laser desorption ionization-time of flight mass spectrometry (MALDI-ToF MS) on an Ultraflex MALDI-ToF/ToF MS instrument (Bruker Daltonics, Germany) equipped with a 337-nm-wavelength nitrogen laser and operated by the MALDI FlexControl software (Bruker Daltonics). A matrix of 9 mg/mL solution of 2,5-dihydroxybenzoic acid (Sigma-Aldrich) in 30% acetonitrile (VWR) was used. All measurements were performed in positive ion, reflector mode with 1,000 shots taken per spectrum.

## Results

### CAZyLingua accurately classifies CAZymes

To comprehensively evaluate its performance, we benchmarked the CAZyLingua RF model against state-of-the-art tools: dbCAN2’s HMM and DIAMOND modules, and the CUPP model [46].

On our three initial test genomes, the CAZyLingua RF model demonstrated F1 scores comparable to the top-performing HMM and CUPP methods (Fig. 2a). It is important to note that the HMM and CUPP models were trained on the entire CAZy database, which may include sequences from our hold-out test set, potentially giving them an inflated score in this comparison. Given this potential for training data overlap, we sought a direct performance comparison against a method based purely on sequence homology, the dbCAN2 DIAMOND tool. We evaluated the receiver operating characteristic (ROC) and precision-recall curves for this head-to-head comparison, using the aggregated results from all three gold-standard genomes (Fig. 2b). The results showed CAZyLingua outperformed dbCAN2’s DIAMOND-based approach. Our model can detect up to 94% of the true CAZymes while maintaining a precision of over 90%, an improvement over the sequence-based approach.

Finally, to ensure our model performs well across a wide range of bacteria and demonstrate robust taxonomic coverage, we solely benchmarked CAZyLingua’s performance on each of 12 gold-standard genomes individually. CAZyLingua maintained consistently high precision, recall, and F1 scores across these varied species (Fig. 2c), confirming the generalizability and effectiveness of our RF-based approach. When checking the micro-averaged classification accuracy of all the families in the test genomes, CAZyLingua predicted 99.6% of the families accurately, while dbCAN2 predicted 98.2% accurately.

### CAZyLingua identifies horizontally-transferred genes as CAZymes

**Fig. 3:**
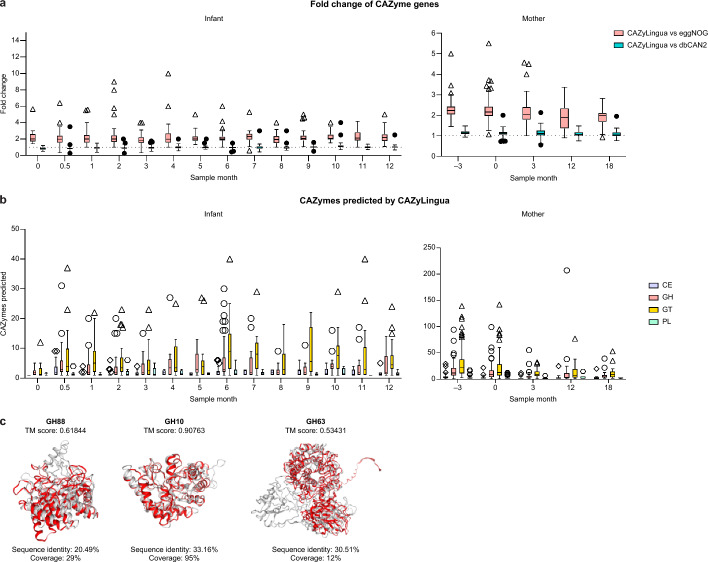
Application of CAZyLingua to metagenomes from paired mothers and infants. **a** Comparison of CAZyLingua to eggNOG and dbCAN2 on a large metagenomics gene catalog from mothers and their infants. Time of the sample is in months relative to childbirth (month 0). Dotted lines represent no fold change. **b** CAZyLingua predicted 27,891 genes that dbCAN2 did not, shown by CAZy class for all infant and maternal samples at each sample month. Boxplots in **a** and **b** show medians and interquartile ranges (IQRs), with whiskers showing ± 1.5 IQR. **c** Predicted structures of proteins from CAZyLingua (red) and the protein embedding nearest neighbor (grey) structurally aligned, with TM scores and BLAST metrics, for GH88, GH10, and GH63

We further tested if CAZyLingua would be able to uncover CAZymes in a gene catalog of microbiome samples from mother/infant pairs collected from late pregnancy to one year of age [39]. We predicted CAZymes using CAZyLingua, alongside eggNOG and dbCAN2, on the entirety of the gene catalog, which contained 2,327,970 genes. CAZyLingua predicted 81,799 CAZymes, while dbCAN2 and eggNOG predicted 77,614 and 38,862 CAZymes, respectively. We stratified the dataset by number of genes per sample, then by sample month, and split the observations by mother and infant. CAZyLingua predicted at least twofold more new genes in maternal and infant metagenomes compared to eggNOG and on average 1.2-fold more new genes than dbCAN2 (Fig. 3a). When examining the predictions made by CAZyLingua, we observed 27,891 unique CAZyme predictions that were not made by dbCAN2. We distinguished each unique CAZyme by CAZyme class within each sample over each sample month and observed that our model predicted many more GTs, followed by GHs, across all the samples in every month (Fig. 3b).

We next focused on a subset of the metagenomic data to specifically look at genes that were found to be horizontally-transferred between a mother/infant pair. A previous study performed a sequence homology (BLASTn) analysis on DNA sequences between maternal and infant metagenomes and identified 977 genes with 100% nucleotide identity that were harbored by both maternal and infant species [39], a portion of which were predicted to function in carbohydrate metabolism. Of the 977 genes, 12 were predicted as CAZymes by our model and either not predicted or predicted as an unknown family within a CAZyme class by dbCAN2.

In order to understand the structural contributions of language models to the general predictions given from ProtT5 and ultimately our pLM classifier, we searched for nearest neighbors between our 12 horizontally-transferred gene embeddings in the CAZy database embeddings using Euclidean distance. After identifying nearest neighbor pairs and extracting the corresponding protein sequences, we computed structural predictions for those proteins using ColabFold [56]. We used FoldSeek [57] to perform a structural alignment between the structures of the predicted protein from CAZyLingua and the nearest protein embedding neighbor in the CAZy database.

CAZyLingua predicted four GHs, including three belonging to the families 88, 10, and 63, that had a high structural homology to their nearest neighbor in the CAZy database (all with a template modeling (TM) score > 0.50, which indicates a same fold between two proteins [60]). In contrast, when evaluating sequence homology (BLASTp) between the amino acid sequences of the three proteins and the nearest neighbor in the CAZy database, we found that between both sets of sequences the sequence identity was lower than 35%, and for GH88 and GH63 the coverage was less than 30% (Fig. 3c). Given these metrics, this suggests that CAZyLingua is able to predict CAZymes incorporating structural homology, despite the lack of any amino acid sequence homology.

**Fig. 4:**
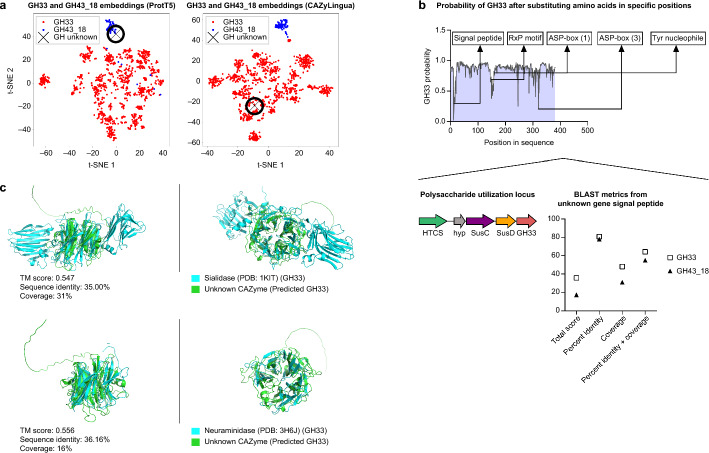
CAZyLingua distinguishes GH33 CAZyme from nearest neighbors of raw ProtT5 embeddings. **a** tSNE of (left) ProtT5 embeddings from the GH33 and GH43_18 families and the CAZyme predicted by CAZyLingua (GH unknown) and (right) a segment of the last layer of CAZyLingua. **b** GH33 protein residues were mutated in a sliding window of ten residues over the entire sequence, and ProtT5 embeddings were generated for each sliding window mutation. Known features were overlaid along sections of the sequence. The probability of the CAZyLingua-predicted classification being a GH33 was calculated for each sliding window mutation (top). The predicted GH mapped to a PUL containing several regulatory elements consistent with a CAZyme (bottom left). BLAST metrics on the predicted GH signal peptide compared with GH33 and GH43_18 sequences (bottom right). **c** Overlays of the predicted GH protein structure generated using ColabFold with a sialidase (top) and a neuraminidase (bottom)

The fourth GH predicted was given the annotation of GH43_18 when evaluating the ProtT5 nearest neighbor, while CAZyLingua classified it as a GH33 (Fig. 4a). We sought to explain if the classification of a GH33 was based on specific features of the unknown CAZyme. We first evaluated the neighborhood of genes around the unknown CAZyme to establish if it exists in a functional polysaccharide utilization locus (PUL). We found several canonical PUL features, including several regulatory elements related to carbohydrate metabolism: a hybrid two-component system (HTCS), TonB-dependent receptor (SusC homolog), and contiguous substrate-binding lipoprotein (SusD homolog) (Fig. 4b). In addition to this unknown enzyme mapping to a PUL, we established the presence of a lipoprotein signal peptide in the enzyme through SignalP [65]. We then explored the link between several functional sites in the GH33 and the corresponding embedding generated by ProtT5. To do so, we created a sliding window of 10 amino acids and created more distant substitutions of the original sequence within that window based on the BLOSUM62 distance. Substituting areas near the signal peptide corresponded to the greatest losses in the CAZyLingua predictive value of a GH33. The first 20 amino acids that correspond to a signal peptide were used in a homology search, and in all BLAST metrics, the signal peptide showed stronger homology to GH33: a combined percent identity and coverage of 64.2% for GH33 and 55.0% for GH43_18, providing stronger evidence for its classification as a GH33 (Fig. 4b).

To determine if there was any structural homology between our unknown CAZyme and the GH33 family, we used ColabFold [56] to fold our protein and ran a structural search with 3D crystal structures found in the PDB25 database using DALI [59]. Our unknown protein had several matches, with two in the top five matches being GH33-like enzymes, namely a neuraminidase and a sialidase. After structurally aligning [60] our unknown structure with the neuraminidase and the sialidase crystal structures, we observed that the predicted GH33 shared significant structural homology (TM score > 0.5) with both. The sequence similarity (BLASTp) between the amino acid sequences pairwise with the unknown protein was again low (sequence identities < 36% and coverages < 31%), demonstrating CAZyLingua’s ability to detect potential remote homologs by structure similarity (Fig. 4c).

### Analysis of enriched CAZymes in inflammatory disease metagenomic gene catalogs

We next focused our attention on applying CAZyLingua to two metagenomic datasets derived from patients with inflammatory and fibrosis-prone diseases: one from 58 IgG4-RD patients and 165 healthy controls [20] and another from 68 CD patients and 34 control subjects [51]. Both of these disease states have unique microbial signatures potentially underlying pathologic mechanisms.

**Fig. 5:**
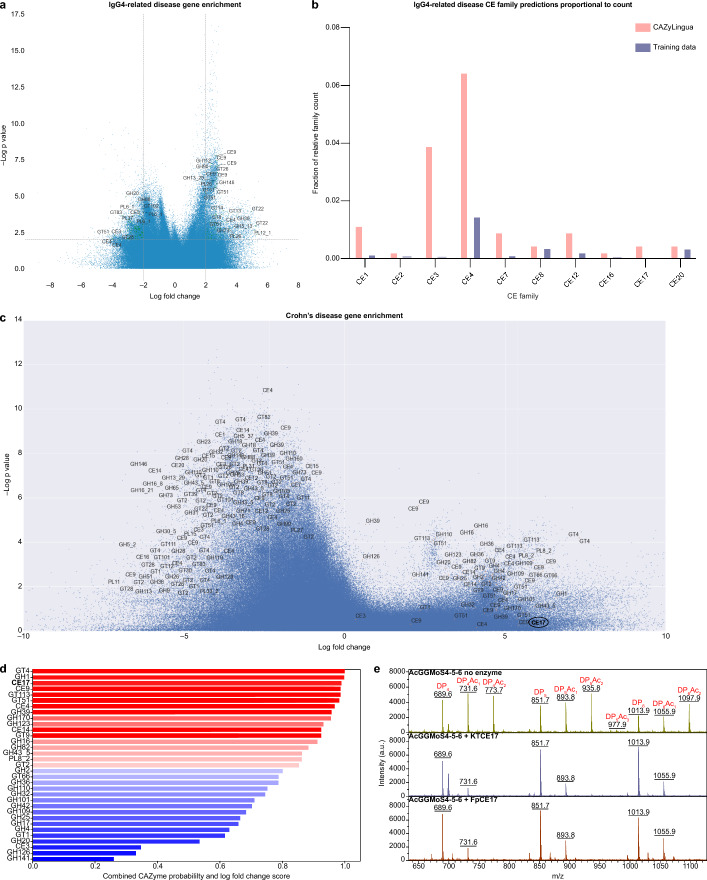
Application of CAZyLingua to CAZymes in metagenomes of patients with inflammatory and fibrosis-prone diseases. **a** Genes enriched and depleted in the gene catalogs of patients with IgG4-RD selected on the fringe of the volcano plot (see Methods for labeling criteria). **b** Predicted CEs in the enriched IgG4-RD gene set, stratified to analyze only the genes CAZyLingua predicted. **c** Genes enriched and depleted in the gene catalogs of patients with CD selected on the fringe of the volcano plot (see Methods for labeling criteria). CE17 is highlighted in the circle. **d** The enriched genes in CD predicted by CAZyLingua only were prioritized based on a combination of the log fold change and the probability of the CAZyme annotation from CAZyLingua. The plot is ordered from the highest fold change and CAZyLingua prediction probability (red) to the lowest fold change and prediction probability (blue). CE17 is highlighted in bold. **e** Functional characterization of CE17 using MALDI-ToF MS. Peaks are labeled by degree of polymerization (DP) and number of acetyl (Ac) groups. The annotated m/z values indicate sodium adducts. Intensity is shown in arbitrary units (a.u.). Both the KTCE17 enzyme (middle) and a previously validated CE17, FpCE17 (bottom, [64]) showed the same activity on a *Ri*GH26-pretreated β-mannan substrate, with disappearance of peaks signifying double and triple acetylated oligosaccharides, and decrease in the intensities of peaks signifying mono-acetylated oligosaccharides (containing 3-O-acetylations) and accumulation of deacetylated oligosaccharides

To investigate disease-associated genes that may be unannotated CAZymes, we first used a linear model against the IgG4-RD gene catalog [62, 66] (Methods) and identified 9,225 genes that were significantly more abundant (two-sided* t*-test, *p* < 1 × 10^–2^, log fold change (FC) > 2) and 7,284 genes that were significantly less abundant (two-sided *t*-test, *p* < 1 × 10^–2^, logFC <  − 2) in IgG4-RD. Among these, CAZyLingua predicted 65 more abundant genes and 87 less abundant genes to be CAZymes (Fig. 5a, Additional file 2: Table S4).

We then broadened our focus to all the CAZymes in the IgG4-RD dataset, irrespective of their significance to disease from the linear model. CAZyLingua predicted 434 CAZymes that dbCAN2 did not. Specifically in IgG4-RD, there was a higher number of CEs that only CAZyLingua predicted. Within the entire CAZy database, CE sequences represent only 4% of all cataloged sequences. The limited number of CE sequences in the dbCAN2 database may explain why the DIAMOND version of dbCAN2, which relies heavily on sequence homology, identifies fewer CE hits compared to other CAZyme classes. In our set of genes predicted by CAZyLingua only, we observed that ~ 34% were CEs. Families of CEs that were particularly represented included CE1, CE3, CE4, and CE12 (Fig. 5b). All of these families share SGNH (Ser-Gly-Asn-His) hydrolase activity, which is a conserved structural feature of the enzymes in these families, suggesting that these enzymes may have low sequence similarity but higher structural similarity within each class [67, 68]. The increase in annotations by CAZyLingua for these specific CE families may be due to the unique structural features of the families that otherwise would be hard to annotate by traditional sequence homology methods.

Following the same procedure, we fit a linear model for a differential gene abundance analysis of CD metagenomes. We stratified genes based on the same criteria. Compared with the IgG4-RD dataset, we noticed a higher proportion of genes were significantly more abundant in disease compared to a healthy state. We observed 3,499 genes that were significantly more abundant compared to 30,125 genes that were significantly less abundant in CD. CAZyLingua predicted 30 more significantly abundant and 569 significantly less abundant CAZymes (Fig. 5c, Additional file 3: Table S5). Given the ~ tenfold difference between more abundant genes in controls versus CD, we observe many more glycan-related pathways associated with health compared to CD.

We focused on the smaller subset of CAZymes that were more abundant (logFC > 0) in CD and identified 51 unique CAZymes that were predicted by CAZyLingua. Of these, we wanted to prioritize any enzymes that CAZyLingua had high confidence in predicting alongside a high fold change in patients with CD. We computed the mean between the logFC percentile (normalized among all the logFC values in the CD dataset) and the probability of the predicted CAZyme family and sorted the results based on descending values (Fig. 5d). Notably, we found a protein predicted to be a CE17 with a high confidence and high logFC in CD that could not be annotated by the dbCAN2 suite. To functionally validate this prediction, we tested the CE17 protein sequence on the known oligosaccharide substrate of the family and confirmed that the enzyme is able to remove acetylations from acetylated manno-oligosaccharides (Fig. 5e).

## Discussion

In this study, we introduced CAZyLingua, a novel approach that leverages pLMs to enhance the accurate classification of CAZymes in metagenomic datasets. Our method mitigates the ongoing challenge of assigning functions to the vast array of unannotated microbial enzymes within these datasets, shedding light on their potential roles in various biological processes. The use of pLMs has emerged as a powerful tool for unraveling protein functions in microbial genomics [27, 28, 34], and our results further emphasize their efficacy in this context. The RF classifier, trained on a large and diverse dataset, demonstrated an improvement in the F1 score by 3 to 6% compared to dbCAN2’s DIAMOND option, while achieving comparable precision and recall to advanced methods such as dbCAN2’s HMM and CUPP. To assess potential overfitting, a post-hoc analysis revealed that only 3.56% of test sequences shared 95% amino acid identity (AAI) with our training data. While this minimal overlap confirms that data leakage is not a significant confounding factor, we recommend that for future applications, users implement a more conservative 90–95% identity cutoff when partitioning training and test sets to ensure the most robust estimate of model generalization.

CAZyLingua’s efficacy is evident in its successful identification of previously undiscovered CAZymes within a longitudinal microbiome dataset of mother/infant pairs. We detected over 27,000 unique putative CAZymes that were missed by dbCAN2. Furthermore, our identification of horizontally-transferred CAZymes between mothers and infants highlights the ability of CAZyLingua to uncover potentially crucial enzymatic functions that traditional sequence homology methods might overlook. When investigating GHs that were not recovered by dbCAN2, we noticed that these GH structures shared low sequence similarity (sequence identity < 40%) to the most homologous protein in the embedding latent space. Our analysis of structural similarities between CAZyLingua-predicted enzymes and GH structures highlights the potential of CAZyLingua to predict enzyme functions based on structural conservation (TM score > 0.5), thereby offering insights into their catalytic roles. We note that these findings are based on structural predictions from ColabFold, not crystal structures or experimentally-validated enzymes. One advantage to our choice of ColabFold as a structural prediction tool is that the process of generating a prediction is heavily dependent on a multiple sequence alignment (MSA) between an unknown sequence and a large reference of sequences. The goal of using ColabFold over popular pLM-based structural prediction tools (e.g., ESM-fold, OmegaFold) was for there to be less of a bias between predictions based on embeddings in a process similar to CAZyLingua and how ProtT5 may be trained versus a standard MSA.

We focused on an example of a horizontally-transferred GH33 that was not predicted by dbCAN2, eggNOG, or a nearest neighbors search using ProtT5 in the CAZy database. Upon using ColabFold to fold this GH33, we performed a sensitive structural search using DALI [59] against experimentally-characterized crystal structures (PDB25) and found the top hits to include other GH33 enzymes (a sialidase/neuraminidase), with significant structural homology (TM score > 0.5, Z score > 2). A recent study examining the early colonization of microbes in a murine model [69] highlights an example of vertical transmission of a GH33 sialidase (NanH) between dams and pups. The NanH gene is triggered by sialylated host glycans and aids in the early colonization of *Bacteroides fragilis*. The putative GH33 discovered by CAZyLingua that was transmitted between a maternal *Alistipes finegoldii* strain and an infant *Alistipes putredinis* strain might exhibit similar properties as NanH and could be part of a mechanism to aid in the establishment of *A. putredinis* in the infant gut. Again, sequence similarity between our putative GH33 and NanH was low (33.93% identity, 26% coverage) despite a similar predicted function, indicating that existing sequence homology methods might have overlooked the putative GH33 as a functional homolog. This highlights the strengths of pLMs as alternative tools to augment functional protein homology discovery.

We then extended the utility of CAZyLingua to metagenomic datasets from patients with CD and IgG4-RD. Both diseases share pathological features of fibrotic lesions despite having distinct clinical presentations. Patients with CD have been shown to have lower microbial diversity and carbohydrate utilization pathways in their gut microbiota [70, 71]. Unique microbial signatures have been strongly associated with IgG4-RD, and those signatures included genes linked to carbohydrate metabolism [20]. Our initial analysis focused on genes that were upregulated in IgG4-RD, where we found a distinct set of CAZymes using CAZyLingua. Investigating the taxonomy of those genes, we found several from *Streptococcus* species that are typically found in the oral cavity. In a previous study [20], many *Clostridium* and typically oral *Streptococcus* species were overabundant in the disease phenotype while *Alistipes* and *Bacteroides* species were depleted. Six of the top 20 (30%) putative CAZymes predicted by CAZyLingua mapped to *Streptococcus mutans,* and we observed that many genes from this microbe were upregulated in disease. We observed enrichment of CEs within this species and postulated that there may be several CAZymes that help *S. mutans* adapt to an ecological niche in the gastrointestinal tract of patients with IgG4-RD.

CEs themselves were sparsely populated in our training dataset for CAZyLingua and similarly in the CAZy database of sequences. Due to the imbalance of this class of enzymes, we postulate that sequence homology may fail to annotate these enzymes. During our training procedure, we used a weighted cross-entropy loss, where the weights were proportional to the number of training examples for a given CAZyme family or subfamily. By allowing a more stringent penalty on incorrectly annotating a rare family, we were able to predict more rare families like CEs through CAZyLingua. Moreover, we functionally validated a CE17 predicted by CAZyLingua, giving us confidence that the predictions made accurately translate to CAZyme activity. Other predictions made by CAZyLingua require experimental validation to ensure true protein functional characterization.

The implications of our findings extend beyond the specific datasets analyzed in this study. CAZyLingua’s demonstrated ability to accurately predict CAZymes has broader implications for deciphering the functional potential of microbial communities. A similar procedure of fine-tuning pLM embeddings can be broadly applied to other enzyme classes and protein domains to aid in functional annotation. As an ever-growing number of metagenomic datasets become available, the incorporation of deep learning tools like CAZyLingua into existing methods offers a promising avenue for comprehensive and accurate functional annotation.

## Conclusions

CAZyLingua represents a novel methodology in the field of CAZyme family annotation. By leveraging pretrained language models, our approach successfully identifies and annotates CAZymes that are often missed by traditional sequence homology methods. The improved accuracy in CAZyme classification, coupled with the ability to uncover novel CAZymes, demonstrates the potential of CAZyLingua to enhance our understanding of microbial communities and their functional capabilities. Our findings in the context of mother/infant microbiome transmission and disease-specific metagenomic datasets highlight the broad applicability of this tool.

## Supplementary Information

Additional file 1.

Additional file 2.

Additional file 3.

## Data Availability

The sequencing dataset generated and analyzed for the IgG4-RD study is available in the NCBI BioProject under PRJNA615162 (https://www.ncbi.nlm.nih.gov/bioproject/ PRJNA615162). Metagenomic sequences for the PRISM IBD cohort used for CD are available via SRA with BioProject number PRJNA400072. Metagenomic sequences for the mother/infant cohorts under the EDIA project are available via SRA with BioProject number PRJNA821542. All embeddings used for training as well as the curated training sequence file dataset can be downloaded at https://gitlab.com/kthurime/cazyclassfier. The version used in this article can be found under release: https://gitlab.com/kthurime/cazyclassfier/-/releases/v1.0. The gene catalogs used in the analysis can be found in Figshare at 10.6084/m9.figshare.26384185. The functionally characterized CE17 DNA sequence can be found under GenBank Accession ID: PQ096848. All of the software required for training and testing and running inference on any protein sequence embedding is available at https://gitlab.com/kthurime/cazyclassfier. All the jupyter notebooks for the analyses generated are available at https://gitlab.com/kthurime/cazyclassfier. A free standing web application can be run at https://marimo.io/@kthurimella/cazylingua.

## Abbreviations

a.u.: Arbitrary unit
Ac: Acetyl
AcGGM: Acetylated galacto-glucomannan
AAI: Average amino acid identity
ASHA: Async Successive Halving
CAZyme: Carbohydrate-active enzyme
CD: Crohn’s disease
CE: Carbohydrate esterase
CUPP: Conserved Unique Peptide Patterns
DP: Degree of polymerization
EDIA: Early Dietary Intervention and Later Signs of β-Cell Autoimmunity
FC: Fold change
FDR: False discovery rate
GH: Glycoside hydrolase
GT: Glycosyltransferase
HDF: Hierarchical data format
HMM: Hidden Markov model
HMO: Human milk oligosaccharide
HTCS: Hybrid two-component system
IgG4-RD: IgG4-related disease
IMAC: Immobilized-metal ion affinity chromatography
IPTG: Isopropyl b-D-thiogalactopyranoside
IQR: Interquartile range
KEGG: Kyoto Encyclopedia of Genes and Genomes
m/z: Mass-to-charge ratio
MALDI-ToF MS: Matrix-assisted laser desorption ionization-time of flight mass spectrometry
MSA: Multiple sequence alignment
NanH: Neuraminidase H
PDB: Protein Data Bank
Pfam: Protein families database
PL: Polysaccharide lyase
pLM: Protein language model
PRISM: Prospective Registry in IBD Study at MGH
PUL: Polysaccharide utilization locus
RF: Random forest
ROC: Receiver operating characteristic
SDS–PAGE: Sodium dodecyl sulfate–polyacrylamide gel electrophoresis
SGNH: Ser-Gly-Asn-His
SRA: Sequence Read Archive
tanh: Hyperbolic tangent
TM: Template modeling
tSNE: T-distributed stochastic neighbor embedding
UHGP: Unified Human Gastrointestinal Protein
