# Supplementary material for: Protein language models uncover carbohydrate-active enzyme function in metagenomics
Source: BMC Bioinformatics. 2025 Nov 26;26:285. doi: 10.1186/s12859-025-06286-y (PMC12659350; doi:10.1186/s12859-025-06286-y)
Supplement: Supplementary file 1 — Additional file 1. [file 12859_2025_6286_MOESM1_ESM.pdf]

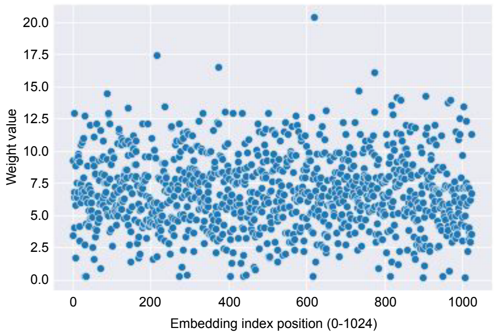

Figure S1. Embedding weights from first layer to next, no interpretable chemical features. We extracted the weights (*W*) from the CAZyLingua multiclass classifier between the input layer and first hidden layer, which is a matrix of dimension 1024x256. After applying a transpose to get W^T we multiplied the two matrices, $W \cdot W^T$ which produced a symmetric matrix, *S* of dimensions 1024x1024. After taking the diag(*S*) we obtained a vector of size 1024, which is the size of the original embedding from ProtT5. We plotted the values in the vector to visualize if there were any features or positions in specific regions of the embedding that are specific to CAZymes.

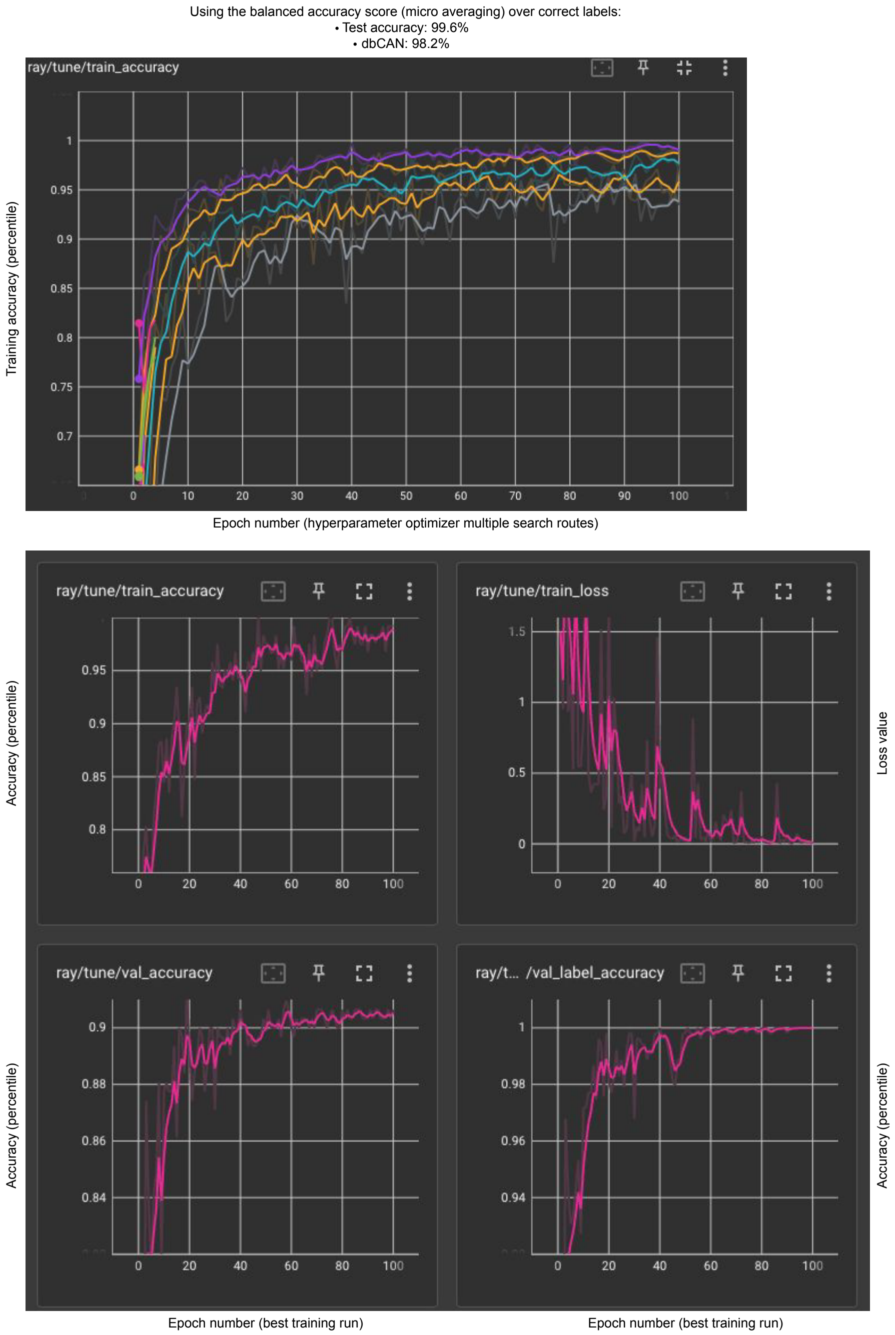

Figure S2. Training runs for finding the best model. RayTune ran 20 models in parallel over each epoch and pruned any models that began to stagnate or have a decline in training accuracy. The models were evaluated on the metric of minimizing training loss, and the model with the minimal loss was stored as a checkpoint. There were 100 epochs over which training occurred, and the metrics were stored and written to a TensorBoard that produced these visualizations.

Group	Genome	Precision	Recall	F1 score	True positive	False positives	True negatives	False negatives
**Original Group**	*Bacteroides thetaiotaomicron*	0.85	0.97	0.91	373	65	4622	11
**Original Group**	*Ruminococcus gnavus*	0.9	0.92	0.91	92	10	3236	8
**Original Group**	*Eggerthella lenta*	0.8	0.94	0.87	33	8	3027	2
**Group 1**	*Roseburia hominis*	0.9	0.91	0.9	174	19	3696	18
**Group 1**	*Streptococcus mutans*	0.83	0.81	0.82	39	8	1848	9
**Group 1**	*Prevotella copri*	0.93	0.95	0.94	87	7	2105	5
**Group 2**	*Faecalibacterium prausnitzii*	0.93	0.94	0.93	74	6	2556	5
**Group 2**	*Lactobacillus acidophilus*	0.93	0.81	0.87	55	4	1801	13
**Group 2**	*Clostridium diolis*	0.96	0.89	0.92	135	6	4773	16
**Group 3**	*Blautia wexlerae*	0.94	0.87	0.9	91	6	4011	14
**Group 3**	*Bifidobacterium breve*	0.97	0.94	0.95	72	2	1804	5
**Group 3**	*Akkermansia muciniphila*	0.88	0.91	0.9	103	14	2242	10

Table S1. Taxonomic hold-out performance. This table contains taxonomic hold-out performance of a model across various bacterial species. It shows precision, recall, and F1 scores as well as counts of true positives, false positives, true negatives, and false negatives for each species, grouped into four categories: Original Group and Groups 1-3.

Hyperparameter	Sampling Method	Sampled Values
Layer 1 Size	Random Choice	(256, 512, 768)
Layer 2 Size	Random Choice	(512, 1024, 1536)
Batch Size	Random Choice	(127, 256, 512)
Learning Rate	Log Uniform Sample	[1e-4 – 1e-2]

Table S2. Hyperparameter tuning. Training epochs over time to pick the model with the best classification accuracy. Using RayTune, we performed a random grid search of different hyperparameter values and tested 20 models in parallel. We picked the model with the best accuracy and used that as the model for all further inference.

Organism	CUPP	HMM dbCAN2	CAZyLingua (RF)	DIAMOND dbCAN2
R. gnavus	0.88	0.91	0.91	0.82
E. lenta	0.4	0.89	0.89	0.68
B. thetaiotaomicron	0.94	0.93	0.91	0.85

Table S3. Benchmarking scores among models. This table compares the performance of different models in classifying CAZymes across three bacterial species. It showcases F1 scores for four different approaches: CUPP, the HMM and DIAMOND modules from dbCAN2, and the RF from CAZyLingua.
